# Postpartum depression symptoms: prevalence, risk factors, and childbirth experiences in Palestine

**DOI:** 10.1186/s12889-024-18829-8

**Published:** 2024-05-20

**Authors:** Batoul Mattar, Niveen M. E. Abu-Rmeileh, Yasmeen Wahdan

**Affiliations:** Institute of Community and Public Health, Birzeit University, P. O. Box 14, Ramallah, West Bank occupied Palestinian territory

**Keywords:** Postpartum depression, Mistreatment, Disrespect, Abuse, Childbirth, Risk factors

## Abstract

**Background:**

Worldwide mothers are suffering from postpartum depression (PPD) which impairs mothers’ well-being, children, and families, and leads to adverse outcomes for mothers and their growing newborns. Low and middle-income countries have a higher prevalence of PPD and limited studies about it. This study assessed the percentage of Palestinian mothers experiencing PPD nationally, identified mothers at a higher risk of PPD, and studied the correlation between PPD and mistreatment during childbirth.

**Methods:**

The study is based on a secondary data analysis from a cross-sectional study in the occupied Palestinian territory (oPt). A total of 745 telephone-based interviews with mothers were done within 2–4 weeks post-childbirth. The Patient Health Questionnaire (PHQ-9) was used as a screening tool for PPD. The Statistical Package for Social Science (SPSS) was used for analyzing the data.

**Results:**

In the context of descriptive epidemiology, we observed that 12.6% of the selected Palestinian mothers experienced PPD, with a higher occurrence of PPD among mothers living in the Gaza Strip, a politically and economically unstable region in Palestine, compared to mothers living in the West Bank (Adjusted Odd Ratio (AOD: 2.2, Confidence Interval (CI): 1.4–3.44). Older mothers were two times more likely to develop PPD compared to young mothers (AOR: 2.03, CI: 1.070–3.84). Mothers who experienced disrespectful behaviors represented by any abuse, negligence, abandonment, ineffective communication, or poor pain management in childbirth settings were more likely to report PPD than those who were not exposed to the disrespect.

**Conclusion:**

A notable percentage of Palestinian mothers were identified as experiencing PPD, especially among mothers who experienced mistreatment in childbirth settings. It is essential to support healthcare providers to improve their practices and attitudes to eliminate mistreatment and abusive behaviors of mothers during childbirth.

**Supplementary Information:**

The online version contains supplementary material available at 10.1186/s12889-024-18829-8.

## Introduction

Childbirth and after childbirth are considered critical and vulnerable periods in any mother’s life due to the physical, emotional, and social changes it carries [1]. After childbirth time, mothers experience rapid hormonal changes that make them vulnerable to mood disturbances; these mood disturbances range from postpartum blues, considered a normal hypersensitivity emotional state. For example, it may be represented by crying easily but is accompanied by a feeling of happiness. Postpartum blues affects 50% of mothers and peaks within three to five days post-childbirth [2]. However, mothers may develop a more intensive mood disturbance called postpartum depression (PPD), a form of major depression that develops within 4–6 weeks post-childbirth and may last six months [2, 3]. PPD has a wide range of symptoms that range from sadness, fatigue, irritability, sleep disturbances, impaired concentration, impaired activity of daily living, and loss of interest to thoughts about maternal suicide or infanticide [2, 3].

PPD is a global problem that affects about 11.9% of mothers all over the world [4]. However, the prevalence of PPD widely varies between countries. This variation can be attributed to several factors, including differences in cultural norms, perceptions of mental distress, associated stigmas, reporting methods, socioeconomic environments, and biological vulnerabilities, all of which influence the occurrence of PPD [5]. For instance, its prevalence was 12% and 8% in the USA and Canada, respectively [6]. The prevalence was high, around 19%, in low- and middle-income countries [7]. The prevalence of PPD in Jordan, which is considered a middle-income and relatively politically stable country, was 25% [8], compared to 28% in Syria, a low-income country currently facing a devastating humanitarian crisis [9]. These two countries are considered neighboring countries to Palestine, where this study took place. Among a few small-scale studies that reported PPD in Palestine, a study conducted in the Nablus district, which utilized different scales and definitions for PPD, showed a PPD prevalence of about 17% [10]. Further, the prevalence of PDD among Palestinian mothers living inside the apartheid wall was 20.8% [11].

Certain risk factors have been shown to affect PPD. For the Arab world, a systematic review showed that mothers with low income were more than 1.5 times more likely to report PPD. Further, PPD was positively associated with young age and low education [12]. In addition, physically abused mothers by their partners were at a higher risk of developing PPD [10]. However, regarding protective factors against PPD, breastfeeding was believed to be one of them as reported in a study from the United Arab Emirates [13].

In addition to the above-mentioned classical factors, nowadays, new studies worldwide are being dedicated to investigating the association between disrespect and abuse in childbirth settings with the mother’s physical and psychological health, including PPD [14–16]. For mothers, childbirth is a special day, an event that is ingrained in the mother’s memory; mothers can recall each healthcare provider’s words and behaviors. Furthermore, the mothers’ experiences are reflected in their feelings and well-being, for example, when they experience respectful childbirth care, they will memorize these good experiences, and these memories enable them to maintain a positive feeling and better self-esteem. However if they experience mistreatment during childbirth, they may develop psychological distress and reach PPD [17].

For example, studies conducted in Brazil revealed that mothers who experienced a particular form of mistreatment, such as physical and verbal abuse during childbirth, were more likely to report PPD [14, 16]. Eventually, mothers who experienced three or more types of mistreatment during childbirth were four times more likely to have PPD than those who did not [14]. Furthermore, the negligence of the healthcare provider to mother during the childbirth increased the mother’s risk of reporting PPD by seven times. In contrast, the presence of supportive care during childbirth by healthcare providers by allowing a childbirth companion was considered a protective factor against PPD; the presence of a childbirth companion decreases the mother’s risk of developing PPD [16].

Regarding the correlation between the mistreatment of mothers during childbirth and PPD, limited literature was available from the Arab region. A study conducted in Iraq showed that women who experience disrespect and abuse during childbirth were at a higher risk of reporting PPD, for instance, they found a positive association between ineffective communication during childbirth and PPD [17]. To our knowledge, the research on PPD and mental health in Palestine was also limited. In the domain of descriptive epidemiology, this study examined the percentage of Palestinian women experiencing PPD, identified mothers at a higher risk of PPD, and studied the correlation between PPD and mistreatment during childbirth. This research aimed to guide evidence-based practices in the Palestinian community and the world to eliminate any factors that may impair mothers’ well-being, children, and families.

## Methods

### Study design

This study was based on secondary data analysis from a cross-sectional study in the occupied Palestinian territory (oPt) that aimed to explore women’s experiences during childbirth in health facilities. The survey was conducted from July 2020 to February 2021, during the COVID-19 pandemic, so telephone-based interviews were used. Each interview was done within 2–4 weeks post-childbirth for women who delivered their babies in five childbirth facilities distributed in the West Bank and the Gaza Strip [18].

## Data collection

### Sampling technique

Recruitment of participants took place in childbirth facilities from three different areas in Palestine: Hebron in the south of the West Bank, Ramallah in the center of the West Bank, and the Gaza Strip. The selection of childbirth facilities was based on the master protocol of the World Health Organization (WHO) multicountry study. The inclusion criteria for these facilities, as per the master protocol, required them to be secondary or tertiary facilities, have a high capacity of ≥ 200 births monthly, and be considered to have a well-defined catchment area.

Based on these inclusion criteria, five hospitals in the selected governorates were eligible for recruitment: one governmental and one non-governmental hospital in both Ramallah and Hebron, along with a governmental Hospital in Gaza.

### Participants

A total of 745 women participated in the study. Mothers were selected based on the following inclusion criteria: (1) ≥ 18 years old; (2) Living within 15 km from the hospital; (3) willing and providing consent to participate; (4) mothers who were admitted for childbirth. Mothers who were excluded from the study were: (1) first-degree relative to someone who works in the selected childbirth facilities; (2) admitted to the facility for reasons other than childbirth; (3) unable to provide consent due to distress, or (4) did not have clear contact channel.

### Recruitment procedure

Each participating hospital assigned one data collector who was a staff member at the facility but worked in a different department. The recruitment process took place before mothers were discharged post-childbirth to their homes, the data collectors followed a screening checklist to determine the eligibility of mothers for recruitment. They then explained the study and its confidentiality process to the mothers, obtaining their oral consent for the community-based interviews. Contact details were also collected before the mothers were discharged to their homes.

Community telephone interviews were carried out by female research assistants who had no prior knowledge of the mothers and were not residents of their respective areas. The telephone interviews were conducted individually with the mothers.

### Ethical consideration

The research design was reviewed by the WHO Research Ethics Review Committee on the 24th of July, 2019, the approval number is A65880. Also, it was reviewed by the Institute of Community and Public Health/ Birzeit University research ethics committee on the 26th of September, 2018, the approval number is 9-2018. All mothers provided oral informed consent after the nature of the investigation had been fully explained. The approval for using oral informed consent was obtained from both the WHO Research Ethics Review Committee and the Institute of Community and Public Health/ Birzeit University research ethics committee. It is important to note that this study did not involve human participants who were under 18 years old. The research design is committed to the principle of the Helsinki Declaration of 1989.

### Instrument/questionnaire

The used survey tool included sections about sociodemographics, obstetric history, screening tools for PPD, and mistreatment during childbirth tool. The survey followed the master survey of the WHO multi-country study on Childbirth Experiences.

### PPD screening tool

The Patient Health Questionnaire (PHQ-9) was used in this study as a PPD screening tool. It’s a quick screening tool for depression. Its items correspond with the Diagnostic and Statistical Manual of Mental Disorders (DSM-IV) criteria for diagnosing depression in general [19]. It is a valid and reliable tool for general depression detection in the Arabian context [20, 21], and its Cronbach’s alpha in this study was 0.857 [21].

### Mistreatment during childbirth in health facilities tool

It is a standardized and validated tool for mistreatment during childbirth in health facilities, it was developed by Bohren and colleagues [22]. The mistreatment types included in this secondary analysis were the only ones that were logical and applicable for all mothers who gave birth by normal vaginal birth or via cesarean section. The included mistreatment types were (1) physical abuse, verbal abuse, stigma or discrimination, (2) negligence and abandonments, (3) poor pain management, (4) ineffective communication, and (5) lack of supportive emotional care from healthcare providers, (6) lack of privacy, and (7) the absence of a childbirth companion. The mistreatment typologies were measured by building a scale for each type from other first-order themes. (Annex A)

### Scales’ reliability

The analysis approach used for assessing reliability and internal consistency involved computing the Cronbach’s alpha coefficient for each scale’s items. (Annex A)

## Statistical analysis

**The dependent variable** was PPD which was measured by using PHQ-9. We used a summed items score of PHQ-9 for PPD diagnosis and a score of ≥ 10 as the cut-off point; Mothers with a summed score of 10 or above were considered to have PPD, and mothers with a score of 9 or less had no depression. This used cut-off point has a sensitivity and specificity of 88% for detecting PPD [23–25].

**The independent variables** were the sociodemographic and obstetric history, which includes age, educational level, employment status, income, gravidity (the number of pregnancies), parity (the number of childbirths), mode of childbirth, current breastfeeding status, and breastfeeding initiation time, besides the mistreatment behaviors in health setting during childbirth.

**The univariate analysis** represented by frequencies and proportions, was used for the total sample and was also conducted separately for the Gaza Strip and the West Bank regions. **Bivariate analysis** was also accomplished by using Chi-Square tests (Pearson Chi-square, Fisher’s Exact Test, and Linear-by-Linear Association) to assess the associations between mothers developing PPD and sociodemographic characteristics, obstetric history, and exposure to mistreatment behavior during childbirth. **Multiple Logistic Regression**, odds ratio, and adjusted odds ratio were used to control for possible confounders like the mother’s age, educational level, region, parity, and mode of childbirth. Data analysis was done using the Statistical Package for Social Science (SPSS), using a confidence level of 95%.

## Results

### Sociodemographic and obstetric characteristics of participant mothers

A total of 745 mothers participated in this study, 475/745 (63.8%) of mothers were from the West Bank and they were equally distributed among Ramallah and Hebron governorates, while 270/745 (36.2%) of mothers were from the Gaza strip. The mean age of participants was 27 years; the youngest mother was 18 years old, while the oldest one was 45. A third (34.4%) (256/745) of respondents attained secondary education, some college, or vocational, while only 161/745 (21.6%) of the mothers hadn’t attained a secondary certificate, with a higher percentage among mothers from the Gaza Strip. Most mothers (647/745) (86.8%) were unemployed outside their homes. In the Gaza Strip, 264/270 (97.7%) of the mothers had an income of less than 2000 New Israeli Shekel (NIS) /month (around 620 $ /month), and none had a monthly income of more than 4,000 NIS/ month (1,240 $/month). While in the West Bank, specifically 81/475 (17.1%) of mothers had a monthly family income of fewer than 2000 NIS/month (620$/month), and only 77/475 (16.2%) had a monthly family income of more than 4000 NIS (1,240 $). The majority of participants, accounting for 542/745 (72.8%), had vaginal childbirth. Most mothers, specifically 693/745 (93%), were current breastfeeders, and 471/745 (65%) of participants initiated breastfeeding within the first hour. Almost a fourth of the mothers (23.9%) (178/745) this was their first pregnancy. About half of the mothers (50.8%) (373/745) had one or two children, as shown in Table 1.

**Table 1:** Distribution of participants’ sociodemographic and obstetric characteristics as total in oPt and split by region

Characteristic	West Bank *N* (%) *n* = 475	Gaza *N* (%) *n* = 270	Total oPt *N* (%) *n* = 745
**Mother’s age (years)**			
< 23	105 (22.1%)	66 (24.4%)	171 (22.9%)
23-29.9	206 (43.4%)	124 (45.9%)	330 (44.3%)
≥ 30	164 (34.5%)	80 (29.6%)	244 (32.8%)
**Educational level**			
Less than secondary education	83 (17.5%)	78 (28.9%)	161 (21.6%)
Secondary education, some college, or vocational	164 (34.5%)	92 (34.1%)	256 (34.4%)
Bachelor’s degree or more	228 (48.0%)	100 (37.0%)	328 (44.0%)
**Marital status**			
Married	474 (99.8%)	268 (99.3%)	742 (99.6%)
Divorced or separated	1 (0.2%)	2 (0.7%)	3 (0.4%)
**Employment**			
No work or student	388 (81.7%)	259 (95.9%)	647 (86.8%)
Employed (full or Part)	87 (18.3%)	11 (4.1%)	98 (13.2%)
Family income (New Israeli Shekel (NIS)/ month)*			
< 2000	81 (17.1%)	264 (97.7%)	345 (46.3%)
2000–2999	202( 42.5%)	5 (1.9%)	207 (27.8%)
3000–3999	115(24.2%)	1 (0.4%)	116 (15.6%)
4000 ≤	77 (16.2%)	0	77 (10.3%)
**Gravidity**			
First pregnancy	104 (21.9%)	74 (27.4%)	178 (23.9%)
2–3	201 (42.3%)	86 (31.9%)	287 (38.5%)
4–5	115 (24.2%)	63 (23.3%)	178 (23.9%)
6≤	55 (11.6%)	47 (17.4%)	102 (13.7%)
**Parity**			
1	118 (24.8%)	84 (31.1%)	202 (27.1%)
2	125 (263%)	49 (18.1%)	174 (23.4%)
3–4	160 (33.7%)	89 (33.0%)	249 (33.4%)
At least 5	72 (15.2%)	48 (17.8%)	120 (16.1%)
**Mode of childbirth**			
Vaginal childbirth	340 (71.6%)	202 (74.8%)	542 (72.8%)
Cesarean section	135 (28.4%)	68 (25.2%)	203 (27.2%)
**Current Breast feeder**			
yes	434 (91.4%)	259 (95.9%)	693 (93.0%)
no	41 (8.6%)	11 (4.1%)	52 (7.0%)
**Time of breastfeeding initiation time**	*n = *457	*n = *263	n = 720
Within 1-hour post-childbirth	306 (67.0%)	165 (62.7%)	471 (65.4%)
More than 1-hour post-childbirth	151 (33.0%)	98 (37.3%)	249 (34.6%)

*: **New Israeli Shekel (NIS)** is a currency used in the occupied Palestinian territories (oPt)

### Post-partum depression (PPD)

#### PPD prevalence and its severity among participants

**Fig. 1:**
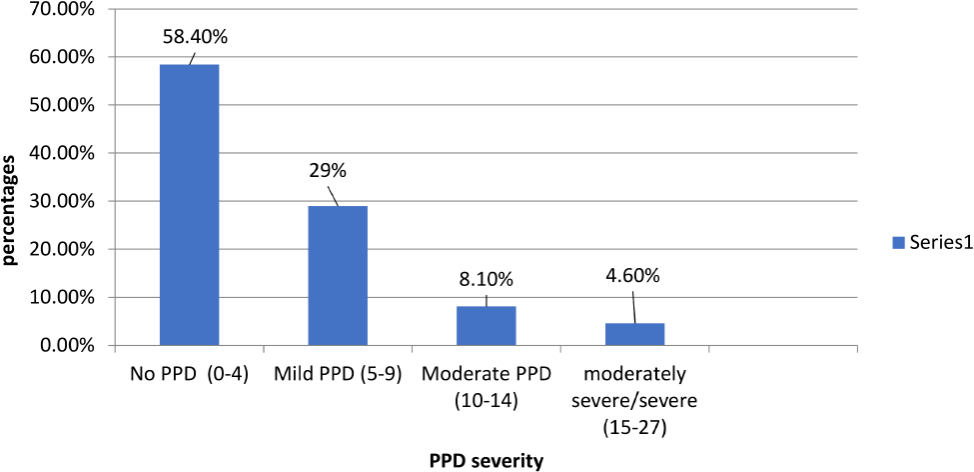
PPD severity distribution based on PHQ-9 score

In the context of descriptive epidemiology, based on using the PHQ-9 score with a cut-off of ten indicating a positive screening for PPD, 94/745 (12.6%) of participants had PPD. We consider the mother depressed if she had moderate PPD or more. Figure 1 shows the distribution of PPD symptoms severity based on PHQ-9 score, 435/745 (58.4%) of mothers had no depression. Among participants, the prevalence of mild, moderate, and moderately severe/ severe depression symptoms was 29% (216/745), 8.1% (60/745), and 4.6% (34/745), respectively.

### PHQ-9 reliability in the Palestinian context

Cronbach’s alpha of PHQ-9 in this study, including all items, was 0.827. As shown in Table 2, only one item, “thought of hurting herself or thought of suicidal” If deleted, Cronbach’s alpha will slightly increase to 0.832. So the used scale, PHQ9, is considered highly reliable because Cronbach’s alpha of 0.9 − 0.7 is considered statistically acceptable [26]. Further, all items have a good correlation since the item-total correlation of > 0.2 was considered statistically acceptable [26].In this study, the inter-item correlations range from 0.679 to 0.329.

**Table 2:** PHQ-9 Scale reliability coefficient and its items in our study

PHQ-9 Items	Mean	Corrected Item-Total Correlation	Cronbach’s Alpha if Item Deleted
A. Little interest or pleasure in doing things	0.503	0.641	0.798
Feeling down, depressed, or despaired	0.542	0.679	0.792
Problems with sleeping, staying asleep, or sleeping a lot	0.836	0.540	0.814
Feeling tired or having little energy	0.923	0.656	0.794
A poor appetite or eating too much	0.776	0.519	0.816
Feeling bad about yourself - or thinking that you are a failure or a burden on your family	0.232	0.638	0.804
Problems focusing on things, such as reading a newspaper or watching TV	0.295	0.559	0.808
Moving or speaking slowly where other people have noticed or the opposite, feeling restless to the extent you start to wander off	0.259	0.406	0.822
The thoughts that you would be better off dying or harming yourself in some way	0.047	0.329	0.832

In the realm of descriptive epidemiology, Table 3 presents the percentage of mothers experiencing PPD based on their sociodemographic characteristics, factors related to pregnancy and childbirth, as well as the childbirth facility. A higher percentage of mothers living in the Gaza Strip, 17.8% (48/270), exhibited PPD compared to mothers living in the West Bank, where the percentage was 9.7% (46/475). PPD percentages were higher among older women and among women with low income (an income of less than 2000 NIS/month) or high-income mothers (an income of more than 4000 NIS/month) than mothers with middle income (2000–4000 NIS/month) in general. However, in the West Bank, PPD percentages were higher among mothers reporting higher income. PPD percentage was high among mothers who were not breastfeeders at the time of the survey and was higher among mothers who gave birth by cesarean secretion than mothers who gave birth by vaginal childbirth. Still, these two correlations (breastfeeding and mode of childbirth) were not statistically significant in the total sample. Noteworthy is that the mode of childbirth was significantly associated with PPD only in the Gaza Strip. Mothers in Gaza who gave birth by cesarean section were significantly more likely to develop PPD than mothers who gave birth by vaginal childbirth. Additionally, the percentage of PPD among mothers who initiated breastfeeding within the first hour of childbirth was significantly less than among mothers who did not initiate it during the first hour. By contrast, educational level, employment status, gravidity, parity, and childbirth facility type were not associated with PPD.

**Table 3:** The prevalence of PPD among participants mothers based on their sociodemographic characteristics, factors related to pregnancy andchildbirth, as well as the childbirth facility. a

Mothers’ characteristics	West Bank (Depressed) *N* = 46	Gaza Strip (Depressed) *N* = 48	Total (Depressed) *N* = 94
**Region of living (Governorate)**			
Ramallah *n* = 237	20 (8.4%)		
Hebron *n* = 238	26 (10.9%)		
Gaza *n* = 270		48 (17.8%)**	
**Mother’s age (Years)** ***n*** ** = 745**			
< 23	7 (6.7%)*	8 (12.1%)	15 (8.8%)*
23-29.9	16 (7.8%)*	25 (20.2%)	41 (12.4%)*
≥ 30	23 (14.0%)*	15 (18.8%)	38 (15.6%)*
**Educational level** ***n*** ** = 745**			
Less than secondary education	5 (6.0%)	14 (17.9%)	19 (11.8%)
Secondary education, some college, or vocational	13 (7.9%)	19 (20.7%)	32 (12.5%)
Bachelor’s degree or more	28 (12.3%)	15 (15.0%)	43 (13.1%)
**Family income (NIS/month),** ***n*** ** = 745**			
< 2000	6 (7.4%)	48 (18.2%)	54 (15.7%)
2000–2999	13 (6.4%)	0	13 (6.3%)
3000–3999	15 (13.0%)	0	15 (12.9%)
4000 ≤	12 (15.6%)*	0	12 (15.6%)*
**Employment** ***n*** ** = 745**			
No work/student	36 (9.3%)	46 (17.8%)	82 (12.7%)
Employed (full or Part)	10 (11.5%)	2 (18.2%)	12 (12.2%)
**Gravidity** ***n*** ** = 745**			
First pregnancy	9 (8.7%)	11 (14.9%)	20 (11.2%)
2–3	19 (9.5%)	18 (20.9%)	37 (12.9%)
4–5	14 (12.2%)	7 (11.1%)	21 (11.8%)
6≤	4 (7.3%)	12 (25.5%)	16 (15.7%)
**Number of babies at home** ***n*** ** = 472**			
One baby only at home	9 (7.7%)	14 (17.1%)	23 (11.6%)
2	11 (8.8%)	13 (26.5%)	24 (13.8%)
3–4	18 (11.3%)	10 (11.2%)	28 (11.2%)
At least 5	8 (11.1%)	11 (22.9%)	19 (15.8%)
**Mode of childbirth** *n* ** = 745**			
Vaginal childbirth	34 (10.0%)	28 (13.9%)	62 (11.4%)
Cesarean section	12 (8.9%)	20 (29.4%)**	32 (15.8%)
**Current Breastfeeder** ***n*** ** = 745**			
yes	40 (9.2%)	45 (17.4%)	85 (12.3%)
No	6 (14.6%)	3 (27.3%)	9 (17.3%)
**Time of breastfeeding initiation time = 745**			
Within 1 h post childbirth	28 (9.2%)	20 (12.1%)	48 (10.2%)
more than 1-hour post childbirth	14 (9.3%)	26 (26.5%)**	40 (16.1%)*
**Involved childbirth facilities**			
Facility 1 *n* = 137	12 (8.8%)		
Facility 2 *n* = 137	16 (11.7%)		
Facility 3 *n* = 270		48 (17.8%)*	
Facility 4 *n* = 100	8 (8.0%)		
Facility 5 *n* = 101	10 (9.9%)		
**Childbirth facility type**			
Governmental *n* = 544			76 (14.0%)
Non-governmental *n* = 201			18 (9.0%)

*P < 0.05, **P < 0.01. PPD was assessed by using the PHQ-9 scale and 10 points as a cut-off point that indicates a positive screening for PPD

a: The percentages in each box represent the percentage of depressed mothers in each category. For example, 6.7% of mothers aged less than 23 years old developed PPD

A previous study in the West Bank and Gaza Strip reported the types and levels of mistreatment during childbirth at a national level [18] (Annex B and Annex C). Table 4 shows the association between reports of mistreatment during childbirth and PPD. It shows that the occurrence of PPD increased among mothers who experienced any physical abuse, verbal abuse, or discrimination, in both the West Bank and Gaza Strip, at a p-value < 0.001. As well as the prevalence of PPD was higher among mothers who experienced poor pain management or negligence and abandonment. Additionally, PPD prevalence was positively and significantly associated with the absence of emotional support from employees during childbirth and ineffective communication between health providers and mothers during childbirth.

While the prevalence of PPD was less among mothers who had a birth companion during the childbirth process (11.8%) (76/646) than mothers who hadn’t a childbirth companion (18.4%) (18/98), the correlation was not significant since the p-value was 0.067. Likewise, as long as a birth companion presents during the labor process the mother was less likely to develop PPD, and this correlation was statistically significant (p-value < 0.01). For example, the prevalence of PPD among mothers who had a birth companion at all stages (before, during, and after childbirth) was 8% (14/175), whereas, its prevalence among mothers who hadn’t a birth companion at any stage was 18.2% (18/99). However, the invasion of privacy during childbirth was not associated with increased reporting of PPD by mothers.

**Table 4:** The association between mistreatment behaviors during childbirth and PPD

Mistreatment types	West Bank (Depressed) a(b)	Gaza Strip (Depressed) a(b)	Total (Depressed) a(b)
**Abuse**			
Any physical abuse, verbal abuse, stigma, or discrimination	15 (19.2%)**	17 (27.4%)*	32 (22.9%)***
verbal abuse	13 (18.6%)**	14 (25.9%)	27 (22.1%)***
**Failure to meet the professional standard of care**			
The poor pain management scale	35 (12.0%)*	30 (24.2%)**	65 (15.7%)**
Negligence and abandonment scale	21 (13.7%)*	28 (21.9%)	49 (17.4%)**
**The poor rapport between women and providers**			
Ineffective Communication	9 (12.0%)	24 (26.7%)**	33 (20.0%)***
Mothers not supported emotionally by employees	11 (9.3%)	24 (26.1%)**	35 (16.7%)*
No Presence of a birth companion during the childbirth process	4 (10.5%)	14 (23.3%)	18 (18.4%)
**Time of birth companion presence (before, during, or post-childbirth)**			
No companion at any stage	4 (10.5%)	14 (23%)	18 (18.2%)**
Companion present at one of the above-mentioned stages	6 (10.3%)	6 (25%)	12 (14.6%)
Companion present at two stages	23 (10.3%)	27 (16.5%)	50 (12.9%)
Companion present at all stages	13 (8.4%)	1 (4.8%)	14 (8.0%)
**Health facility conditions and constraints**			
Absence of Privacy tool (Were curtains, dividers, or other measures not used to provide you with privacy from other patients, family members of patients, health workers, or employees	7 (20.0%)*	1 (5.9%)	8 (15.4%)

^*^P < 0.05, ** *P* < 0.01, ***P < 0.001

a: The counts mentioned in the table represent the number of mothers who developed postpartum depression and experienced mistreatment in childbirth settings

b: The percentages used represent the prevalence of PPD among mothers who only experience certain mistreatment behaviors in childbirth settings

Table 5 shows the odds of having PPD according to the mother’s sociodemographic characteristics, mother obstetric history, and experiencing mistreatment behaviors during childbirth after doing adjustments for possible confounders. Mothers living in the Gaza Strip were 2.2 times more likely to develop PPD than those living in the West Bank by adjusting for age, education, and parity (Adjusted Odds Ratio (AOR): 2.2, Confidence Interval (CI): 1.4–3.44). Older mothers (Mothers aged 30 years and above) were two times more likely to develop PPD compared to young mothers (aged less than 23 years old) when adjusting for the region (AOR: 2.03, CI: 1.070–3.84). Mothers who didn’t initiate breastfeeding within the first hour of childbirth were at a higher risk of developing PPD than mothers who did (Odds Ratio (OR):1.69, CI: 1.07–2.65), But, when controlling for possible confounders like region, age, education, mode of delivery and parity, this correlation no longer exists (AOR:1.57, CI: 0.87–2.85).

Regarding mistreatment during childbirth and PPD, after adjusting for possible confounders such as region, age, education, parity, and mode of childbirth, the results indicate that mothers who experienced any form of abuse (physical, verbal, stigma, or discrimination) were 2.73 times more likely to report PPD than mothers who didn’t (AOR: 2.73, CI: 1.675–4.48). Similarly, mothers who experienced negligence were 1.85 times more likely to report PPD than mothers who didn’t (AOR: 1.85, CI: 1.18–2.9), those who experienced ineffective communication were 2.06 times more likely to report PPD than mothers who didn’t experience ineffective communication(AOR: 2.06, CI: 1.25–3.38), and those who received poor pain management were 2.1 times more likely to report PPD than mothers who didn’t (AOR: 2.1, CI: 1.27–3.49). These findings suggest a significant association between mistreatment during childbirth and the likelihood of experiencing PPD.

Mothers who didn’t have a childbirth companion at any stage of childbirth were 2.5 times more likely to develop PPD than mothers who had a childbirth companion at all childbirth stages (OR = 2.56, 95% CI:1.2–5.4), but when adjustment for possible confounders, this correlation was not statistically significant (AOR = 1.77, 95% CI:0.79-4). Also when controlling for possible confounders, lack of emotional support from healthcare providers during childbirth was not associated with reporting PPD by mothers.

**Table 5:** PPD Adjusted and unadjusted odds ratios at 95% confidence intervals by selected factors

Variable	Unadjusted OR	95% CI	Adjusted OR	95% CI
**Demographic**				
**Age** ^**1**^ **(years)**				
< 23	Reference (Ref)		Ref	
23-29.9	1.48	0.79–2.75	1.50	0.80–2.80
≥ 30	1.92*	**1.02–3.60**	2.03*	**1.07–3.84**
**Educational level** ^**2**^				
Less than secondary education	0.89	0.50–1.58	0.85	0.47–1.55
Secondary education, some college, or vocational	0.95	0.58–1.55	1.03	0.62–1.71
Bachelor’s degree or more	Ref		Ref	
**Employment** ^**3**^				
No work/student	Ref		Ref	
Employed (full or Part)	0.96	0.50–1.84	0.99	0.48–2.06
**Region** ^**4**^				
West Bank	Ref		Ref	
Gaza	2.02*	**1.30–3.12**	2.20*	**1.40–3.44**
**Governorate** ^**4**^				
Ramallah	Ref		Ref	
Hebron	1.33	0.72–2.46	1.43	0.76–2.68
Gaza	2.35*	**1.35–4.08**	2.65*	**1.5–4.69**
**Obstetric history**				
**Parity** ^**5**^				
1	Ref		Ref	
2	1.22	0.66–2.26	1.20	0.63–2.30
3–4	0.97	0.54–1.74	0.68	0.34–1.37
At least 5	1.44	0.75–2.77	0.83	0.35–1.98
**Mode of childbirth** ^**6**^				
Vaginal childbirth	Ref		Ref	
Cesarean section	1.45	0.91–2.30	1.28	0.78–2.08
**Breastfeeding initiation time** ^**7**^				
Within 1-hour post-childbirth	Ref		Ref	
More than 1-hour post-childbirth	1.69*	**1.07–2.65**	1.57	0.87–2.85
**Mistreatment practices during childbirth** ^**7**^				
**Any abuse**	2.65*	**1.65–4.27**	2.73*	**1.67–4.48**
**Poor pain management**	1.97*	**1.23–3.15**	2.10*	**1.27–3.49**
**Negligence and abandonment**	1.97*	**1.27–3.04**	1.85*	**1.18–2.90**
**Ineffective communication**	2.21*	**1.38–3.53**	2.06*	**1.25–3.38**
**Lack of emotional support from employee**	1.61*	**1.03–2.54**	1.55	0.97–2.48
**Companion time of the present**				
No companion at any stage	2.56*	**1.2–5.4**	1.77	0.79- 4.00
Companion present at one of the childbirth stages	1.97	0.87–4.48	1.60	0.68–3.78
Companion present at two stages	1.7	0.91–3.17	1.24	0.63–2.44
Companion present at all stages	Ref		Ref	
**Privacy wasn’t respected during checks, exams, and treatments (subjective)**	2.15	0.95–4.88	2.30	0.98–5.40
**Absence of privacy tool**	1.28	0.58–2.80	1.34	0.60–2.98

^1^ Adjusted for the region, ^2^ Adjusted for region and age, ^3^ Adjusted for the region, age, education, and parity, ^4^ adjusted for age, education, and parity, ^5^ adjusted for age, education, and parity, ^6^ adjusted for region, age, education, and parity, ^7^ adjusted for region, age, education, mode of delivery and parity.*p value < 0.05

## Discussion

As far as we know, this study is one of the limited studies measuring postpartum depression (PPD) in the occupied Palestinian territory (oPt). Besides one of few studies investigating the association between PPD and the mistreatment of mothers in childbirth settings at a national level. This study revealed the occurrence of PPD in the West Bank and the Gaza Strip and its possible risk factors. In the light of descriptive epidemiology, we observed that 12.6% of mothers in the oPt had PPD, with a double occurrence among mothers living in the Gaza Strip compared to those living in the West Bank. Older women were two times more likely to develop PPD than young mothers. This study also showed that mothers who experienced disrespectful childbirth care were more likely to report PPD than those who were not exposed to any form of disrespect.

The observed percentage of mothers experiencing PPD in this study was 12.6%, which was relatively high compared to the developed countries, 12% and 8% in the US and Canada, respectively [6], and relatively low compared to the reported pooled prevalence in the Middle East (ME) (27%). In ME, the prevalence in ME range was between (The highest: 56 %) in Kermanshah City, Iran, and (the lowest: 10%) in the United Arab Emirates which is a region of high income [12]. The percentage of mothers identified with PPD in this study was also relatively lower than PPD prevalence in the neighboring countries, namely Jordan and Egypt, in which PPD ranges between 20–22% [7]. This variation in results can be attributed to the socioeconomic, cultural, and biological variance or the use of a different screen tool for PPD [5]. Almost all of the research in ME used the Edinburgh Postnatal Depression Scale (EPDS) while our study used PHQ-9. But, compared to a study that used the same screening tool (PHQ-9), a study was conducted in Ghana, a low-income country in Africa, the PPD prevalence there was 7%, so the prevalence among this study participants shows a relatively higher PPD compared to their findings [27].

The relatively high percentage of mothers identified with PPD in oPt can be due to prolonged political and economic stability in oPt, since another study in oPt emphasized that the mental health of Palestinians is inextricably intertwined with political, economic, and social factors [28]. The effect of political and economic instability also can be better explained by the regional variation in oPt, the observed percentage of mothers with PPD in the Gaza Strip is 2.2 times more than in the West Bank in this study. That can be explained by the fact that the Gaza Strip is the most suffering area in oPt politically and its residents have suffered a siege since 2006 and multiple targeted attacks. All of these latter factors result in devastating consequences on the economic situation of the Gazan people, represented by high poverty rates and high unemployment rates [29]. Furthermore, Gazan people lack human basic needs; their movement is restricted, and they feel unsecured and traumatized, so, mothers residing in this conflict zone are vulnerable to mental distress and suffer cumulative stressors [28].

Regarding the impact of Israel’s Occupation on the mistreatment of mothers in childbirth facilities, researchers found that the main underlying reason for this mistreatment is the long-term political and financial instability effect on health facilities. This is manifested by a shortage of equipment, poor infrastructure, understaffing, limited staff development, and an overwhelming workload in childbirth settings. These factors negatively affect the well-being of the staff and consequently hinder their ability to provide respectful, satisfactory, and safe care to mothers during childbirth [30, 31].

Furthermore, the condition of health settings is worse in the Gaza Strip, as Gaza experiences a more deteriorated and prolonged socio-economic situation, conflict, and siege compared to the West Bank. In addition to the above-mentioned consequences of wars on Palestinian health facilities, health facilities in Gaza have also been adversely affected by impaired access to medical equipment, limited training opportunities, and frequent electricity cuts. These challenges have significantly weakened health facilities, the services provided, and the overall health of the population in Palestine and especially Gaza [32].

However, mothers in the Gaza Strip, who experienced traumatic war events characterized by losses, material destruction, and horrors, were at a higher level of mental distress during pregnancy or post-childbirth. In turn, this devastating effect on the mother’s mental well-being mediates the negative impact of war on infant development, for instance, children of mothers mentally distressed due to war were at higher risk for motor developmental delay [33].

Regarding the mistreatment of mothers in childbirth settings, a previous study in the West Bank and Gaza Strip reported the types and levels of mistreatment during childbirth [18]. Our study documented the association between mistreatment types during childbirth and PPD. Our study findings were consistent with similar studies conducted in Brazil and Iraq where women who experienced disrespect and abuse during childbirth were at a higher risk to report PPD regardless of the difference in settings [17, 34].

Regarding negligence and abandonment during childbirth, our findings were congruent with Souza and colleagues’ findings in that being neglected as a mother during childbirth positively correlates with an increased likelihood of developing PPD [16]. Moreover, the findings of the Iraqi study were consistent with ours, for we found a positive association between ineffective communication during childbirth and PPD [17].

Regarding childbirth companions, our study findings and others’ findings in Brazil revealed that childbirth companions worked as a protective factor against PPD [16], This may be because the presence of a childbirth companion protects the mother from abusive behavior from healthcare providers and achieves a better childbirth experience [35], and this positive and respectful childbirth experience reflects on the mother’s mental well-being positivity by less PPD prevalence since our findings show that respectful childbirth experience decreased the mother likelihood to develop PPD.

Although poor economic status was strongly associated with PPD in the Arab world [12], a similar association was not detected in this study. This might be explained by the fact that the study was conducted during the COVID-19 Pandemic which had economic implications for the whole population [29, 36]. Moreover, there was a huge difference in the reported monthly income between women living in the West Bank which was higher compared to women living in the Gaza Strip. Therefore, a further detailed economic study is required in Gaza, considering the employment rate, income, and other economic factors.

In contrast to other studies, our study showed a different trend regarding younger mothers and their tendency towards PPD. In our study, mothers aged 30 years old or above were at a higher risk of developing PPD than younger mothers aged 18 to 23 years old. This difference can be attributed to the fact that our ‘younger’ age group comprised mothers aged 18 to 23, excluding adolescent mothers under the age of 18, who are known to face greater challenges in caring for a newborn and thus have a higher risk for PPD, as discussed in other studies [37].

### Study strengths and limitations

This study was conducted at the national level including both the Gaza Strip and the West Bank. The study had minimum recall bias as most mothers were interviewed within 2–4 weeks post-childbirth, which is also an optimal time to detect PPD. One limitation of this study is the use of the PHQ-9, which is a non-definitive diagnostic tool for PPD; While a positive result on the PHQ-9 indicates a potential presence of PPD, the study did not employ a clinical evaluation by healthcare professionals to confirm the diagnosis. In addition, it is important to note that the focus of the study was to study the childbirth process and hence other prenatal and antenatal factors that may affect PPD were not included. For instance, the challenge of not excluding mothers with prenatal depression hinders a precise understanding of the association between mistreatment during childbirth and PPD, as prenatal depression is considered a risk factor for PPD. Another limitation of this study is that all participants were recruited from the central and southern regions of the West Bank and the Gaza Strip. This limitation restricts the generalizability of the results to the entire oPt. Additionally, the sample size in the Gaza strip did not reach the minimum required to achieve a 95% confidence interval. Lastly, this study is not able to explore economic influences on maternal well-being post-childbirth, given it was conducted during the COVID-19 Pandemic, which had widespread economic implications for the entire population.

## Conclusion

We observed a significant propotion of Palestinian mothers experiencing postpartum depression (PPD), especially among mothers who experienced mistreatment in childbirth settings, or who live in politically and economically unstable regions.

To improve maternal health, systemic and individual efforts are needed to prevent disrespect and abusive behavior in childbirth settings to ensure high-quality, dignified, and respectful maternity care. To afford respectful care for Palestinian mothers during childbirth, more investments in the healthcare system are needed to make it well-equipped. Furthermore, it is necessary to support healthcare providers, who are part of the suffering Palestinians from hopelessness, fear, fatigue, and inconsistent salaries. Respectful care can be done through less workload, incentives, motivation, and training for healthcare providers to eliminate disrespect and abusive behaviors and enhance effective communication with mothers. And what is most important and effective in preventing and treating PPD, is adopting political, social, and cultural approaches, not a medical approach only, because Palestinians were also suffering from cumulative exposure to unpleasant political, social, and economic pressure, which affects the mental well-being of all Palestinians, mothers, and their families.

### Electronic supplementary material

Below is the link to the electronic supplementary material.

Supplementary Material 1

## Data Availability

The datasets used and analyzed during the current study are available from the corresponding author upon reasonable request.

## Abbreviations

PPD: Postpartum Depression
oPt: Occupied Palestinian territory
PHQ-9: Patient Health Questionnaire
WHO: World Health Organization
SPSS: Statistical Package for Social Science
APA: American Psychiatric Association
DSM-IV: Diagnostic and Statistical Manual of Mental Disorders (Fourth Edition)
NIS: New Israeli Shekel
$: Dollar
OR: Odds Ratio
AOR: Adjusted Odds Ratio
CI: Confidence Interval
ME: Middle East
EPDS: Edinburgh Postnatal Depression Scale
AI: Artificial Intelligence
UNRWA: United Nations Relief and Work Agency for Palestine Refugees in the Neat East
