# Supplementary material for: Postpartum depression symptoms: prevalence, risk factors, and childbirth experiences in Palestine
Source: BMC Public Health. 2024 May 20;24:1357. doi: 10.1186/s12889-024-18829-8 (PMC11107043; doi:10.1186/s12889-024-18829-8)
Supplement: Supplementary file 1 — Supplementary Material 1 [file 12889_2024_18829_MOESM1_ESM.docx]

**Annexes:**

**Annex A:** This study scales developments

**Abuse scale:**

**The physical abuse** scale was developed based on experiencing any form of first-order themes including pinch, kick, slap, punch, beating with a tool, muzzle, tying mother to the hospital bed, forcefully holding mother on the bed, forcefully placing pressure on mother abdominal area before giving birth, or any other physical abuse mentioned by mothers. This scale was recorded to be a binary variable ( no physical abuse or at least experiencing one form of physical abuse), this scale has weak reliability, and Cronbach's alpha was 0.018.

**The verbal abuse** scale was developed based on experiencing any form of first-order themes including screaming, insulting, telling the mother off, ridiculing or making fun of the mother, commenting negatively on the mother's physical appearance, commenting negatively on the baby's appearance, commenting negatively on mother's sexual activity, threatening a mother with a medical procedure (such as a vaginal wound or a C-section, threatening the mother with physical violence, a threatening mother with having or her child having poor outcome, a threatening mother with withholding/stopping care for mother or her child, blaming mother, silence mother by (hush), or Other forms of abuse. This scale was recorded to be a binary variable ( no verbal abuse or at least experiencing one form of verbal abuse), this scale has accepted reliability, Cronbach's alpha was 0.585, but if deleted the items of “Did any of the hospital staff or workers comment negatively regarding your sexual activity?” it improved to 0.588, especially no any mother in our study has exposed to this theme of verbal abuse so we deleted it, also we deleted the items of “Did any of the hospital staff or hospital workers comment negatively on your child’s appearance (such as their outer appearance, gender, or other aspects of the child”, since its deletion improved the scale reliability. The recommended verbal scale contained only 12 items without above mentioned two themes since it has a better Cronbach's alpha of 0.592.

**The stigma scale** developed based on if health workers comment negatively about the mother’s ethnic origin, lineage, village/ clan, culture, religion, age, marital status, level of education, economic status, or HIV status. This scale was recorded to be a binary variable ( no stigma or at least experiencing one form of the above-mentioned stigma theme), this scale has weak reliability, with Cronbach's alpha -0.003.

**Any abuse scale** developed as a binary variable (yes/no) based if the mother experienced any themes of abuse mentioned above (physical, verbal, or stigma), this scale abuse Cronbach alpha: 0.197.

The typology of “**Failure to meet a professional standard of care”** has many themes, the first one is the negligence and abandonment scale which had constructed based on the following first-order themes:

1. Mother felt ignored by health workers

2. Mother felt neglected by health workers

3. Mother felt that her presence was an annoyance to health workers

4. Mother waited a long period before health staff acknowledged or prepared her in the hospital for labor

5. absence of health workers at the time of the child's arrival

**The negligence and abandonment scale** developed as a binary variable, ( no theme of negligence and abandonment or at least one form of negligence or abandonment, Cronbach's alpha with all above-mentioned theme 0.727, but It is 0.794 if the Items of “presence of health worker at the time of baby arrival” was deleted, so we used the modified scale which only contains the first 4 themes mentioned above.

**Poor pain management** developed as a binary variable, ( good pain management, or poor pain management which is represented by either being deprived of pain killer, ordering pain killer, or both.

**The typology of poor rapport between mothers and health providers** is measured by two second-order themes, the first is Lack of Supportive care which is measured by the presence of a birth companion at any stage of the labor process, by having a companion while staying in hospital or mother’s feeling that health workers emotionally supported. The second was the ineffective communication scale which developed as a dichotomous variable ( effective communication, not effective communication) it considers effective communication if the health workers kindly listened and respond to the mother's concerns and questions while she was in the childbirth setting, not effective if health workers didn't listen or didn’t respond to mother concern and questions, Cronbach's alpha for effective communication 0.896.

**Annex B: The prevalence of mistreatment during childbirth among Palestinian mothers.**

Any physical abuse, verbal abuse, discrimination, or stigma was reported by 140/745 (18.8%) of mothers, and it has a slightly higher prevalence in the Gaza Strip (62/270) (23%) compared to the West Bank (78/745) (16.4%). The most common form of abuse was verbal abuse which was reported by 122 /745 (16.4%) of mothers with a slightly higher prevalence in the Gaza Strip around (20%) (53/270) than in the west bank (14.5%) 69/475). Physical abuse and Stigma or discrimination were reported by 25/745 (3.4%) of mothers and 5/745 (0.7%) of mothers respectively, with a higher prevalence of physical abuse in the Gaza Strip (5.2%) (14/270). Regarding failure to meet the professional standard of care, 415/740 (56.1%) of mothers had pain and ordered pain relief or has been deprived of pain relief while they were in the hospital. Also, 281/745 (37.7%) of participant mothers had been neglected and abandoned by health care providers, it has a higher prevalence in the Gaza Strip (47.4%) (128/270) compared to the West Bank (32.3%) (153/475). For the poor rapport between mothers and health providers, 165/736 (22.4%) of mothers had been communicated ineffectively, and the prevalence of ineffective communication in the Gaza Strip (34%) (90/265) was double its prevalence in the West Bank (16%) (75/471). 98/744 (13.2%) of mothers hadn’t a companion during the childbirth process, the percentage was higher in the Gaza Strip (22.2%) (60/270) compared to the West Bank (8%) (38/744). while 210 /745 (28.2%) of mothers hadn’t received emotional support from employees. Regarding privacy, 35/745 (4.7%) of mothers feel that their privacy wasn’t respected during checks, exams, and treatments, while 52/743 (7%) of mothers reported a loss of privacy tools like curtains.

**AnnexC:** The Prevalence of mistreatment during childbirth split by the West Bank and Gaza Strip.

| Mistreatment types | Total | The West bank # (%) | The Gaza strip # (%) |
| --- | --- | --- | --- |
|  | 745 | 475 | 270 |
| Abuse |  |  |  |
| Any physical abuse, verbal abuse, stigma, or discrimination (Yes) | 140 (18.8%) | 78 (16.4%) | 62 (23%) |
| Physical abuse | 25 (3.4%) | 11 (2.3%) | 14 (5.2%) |
| verbal abuse (new scale) | 122 (16.4%) | 69 (14.5%) | 53 (19.6%) |
| Stigma or discrimination | 5 (0.7%) | 4 (0.8%) | 1(0.4%) |
| Failure to meet the professional standard of care |  |  |  |
| Poor pain management** | 415 (56.1%) | 291 (61.3%) | 124 (46.4%) |
| Negligence and abandonment scale (4 items only) | 281(37.7%) | 153 (32.2%) | 128 (47.4%) |
| The poor rapport between women and providers |  |  |  |
| Ineffective Communication ** | 165 (22.4%) | 75 (16%) | 90 (34%) |
| Time of birth companion presence(before, during, or post-childbirth)* |  |  |  |
| No companion at any stage | 99 (13.3% | 38 (8%) | 61 (22.2%) |
| Companion present at one of the above-mentioned stages | 82 (11%) | 58 (12.2%) | 24 (8.9%) |
| Companion present at two stages | 388 (52.2%) | 224 (47.2%) | 164 (60.7%) |
| Companion present at all stages | 175 (23.5%) | 154 (32.5%) | 21 (7.8%) |
| The mother is not supported emotionally by the employee | 210 (28.2%) | 118 (24.8%) | 92 (34.1%) |
| Health Facility Culture, condition, and Constraints |  |  |  |
| The mother felt that her privacy wasn’t respected during checks, exams, and treatments. | 35 (4.7%) | 23 (4.8%) | 12 (4.4%) |
| Lack of resources |  |  |  |
| loss of Privacy tools (Were curtains, dividers, or other measures used to provide the mother with privacy from other patients, family members, health workers, or employees)* | 52 (7%) | 35 (7.4%) | 17 (6.3%) |

**Key: *:** one or two missed cases, **: 5-9 missed cases.

**Annex D: STROBE Checklist:**

STROBE Statement—a checklist of items that should be included in reports of observational studies

|  | Item No | Recommendation | Page  No |
| --- | --- | --- | --- |
| **Title and abstract** | 1 | (*a*) Indicate the study’s design with a commonly used term in the title or the abstract | 2 |
|  |  | (*b*) Provide in the abstract an informative and balanced summary of what was done and what was found | 2 |
| Introduction | | | |
| Background/rationale | 2 | Explain the scientific background and rationale for the investigation being reported | 3-5 |
| Objectives | 3 | State specific objectives, including any prespecified hypotheses | 5 |
| Methods | | | |
| Study design | 4 | Present key elements of study design early in the paper | 6 |
| Setting | 5 | Describe the setting, locations, and relevant dates, including periods of recruitment, exposure, follow-up, and data collection | 6 |
| Participants | 6 | (*a*) *Cohort study*—Give the eligibility criteria, and the sources and methods of selection of participants. Describe methods of follow-up  *Case-control study*—Give the eligibility criteria, and the sources and methods of case ascertainment and control selection. Give the rationale for the choice of cases and controls  *Cross-sectional study*—Give the eligibility criteria, and the sources and methods of selection of participants | 6 |
|  |  | (*b*) *Cohort study*—For matched studies, give matching criteria and number of exposed and unexposed  *Case-control study*—For matched studies, give matching criteria and the number of controls per case |  |
| Variables | 7 | Clearly define all outcomes, exposures, predictors, potential confounders, and effect modifiers. Give diagnostic criteria, if applicable | 7-9 |
| Data sources/ measurement | 8* | For each variable of interest, give sources of data and details of methods of assessment (measurement). Describe comparability of assessment methods if there is more than one group | *7-9, AnnexA* |
| Bias | 9 | Describe any efforts to address potential sources of bias | 6 |
| Study size | 10 | Explain how the study size was arrived at | 6 |
| Quantitative variables | 11 | Explain how quantitative variables were handled in the analyses. If applicable, describe which groupings were chosen and why | 7-9 |
| Statistical methods | 12 | (*a*) Describe all statistical methods, including those used to control for confounding | 9 |
|  |  | (*b*) Describe any methods used to examine subgroups and interactions |  |
|  |  | (*c*) Explain how missing data were addressed |  |
|  |  | (*d*) *Cohort study*—If applicable, explain how loss to follow-up was addressed  *Case-control study*—If applicable, explain how matching of cases and controls was addressed  *Cross-sectional study*—If applicable, describe analytical methods taking account of sampling strategy |  |
|  |  | (*e*) Describe any sensitivity analyses |  |

| Results | | | |
| --- | --- | --- | --- |
| Participants | 13* | (a) Report numbers of individuals at each stage of study—eg numbers potentially eligible, examined for eligibility, confirmed eligible, included in the study, completing follow-up, and analysed | 10 |
|  |  | (b) Give reasons for non-participation at each stage |  |
|  |  | (c) Consider use of a flow diagram |  |
| Descriptive data | 14* | (a) Give characteristics of study participants (eg demographic, clinical, social) and information on exposures and potential confounders | 10-13 annex A, annex B |
|  |  | (b) Indicate number of participants with missing data for each variable of interest |  |
|  |  | (c) *Cohort study*—Summarise follow-up time (eg, average and total amount) |  |
| Outcome data | 15* | *Cohort study*—Report numbers of outcome events or summary measures over time |  |
|  |  | *Case-control study—*Report numbers in each exposure category, or summary measures of exposure |  |
|  |  | *Cross-sectional study—*Report numbers of outcome events or summary measures | *Tables 1-5* |
| Main results | 16 | (*a*) Give unadjusted estimates and, if applicable, confounder-adjusted estimates and their precision (eg, 95% confidence interval). Make clear which confounders were adjusted for and why they were included | 10-25 |
|  |  | (*b*) Report category boundaries when continuous variables were categorized |  |
|  |  | (*c*) If relevant, consider translating estimates of relative risk into absolute risk for a meaningful time period |  |
| Other analyses | 17 | Report other analyses done—eg analyses of subgroups and interactions, and sensitivity analyses |  |
| Discussion | | | |
| Key results | 18 | Summarise key results with reference to study objectives | 26 |
| Limitations | 19 | Discuss limitations of the study, taking into account sources of potential bias or imprecision. Discuss both direction and magnitude of any potential bias | 29 |
| Interpretation | 20 | Give a cautious overall interpretation of results considering objectives, limitations, multiplicity of analyses, results from similar studies, and other relevant evidence | 26-28 |
| Generalisability | 21 | Discuss the generalisability (external validity) of the study results | 26-29 |
| Other information | | | |
| Funding | 22 | Give the source of funding and the role of the funders for the present study and, if applicable, for the original study on which the present article is based | 31 |

*Give information separately for cases and controls in case-control studies and, if applicable, for exposed and unexposed groups in cohort and cross-sectional studies.

**Note:** An Explanation and Elaboration article discusses each checklist item and gives methodological background and published examples of transparent reporting. The STROBE checklist is best used in conjunction with this article (freely available on the Web sites of PLoS Medicine at http://www.plosmedicine.org/, Annals of Internal Medicine at http://www.annals.org/, and Epidemiology at http://www.epidem.com/). Information on the STROBE Initiative is available at www.strobe-statement.org.
